# Editorial: Selective Controls on Microbial Energy Metabolisms: From the Microscale to the Macroscale

**DOI:** 10.3389/fmicb.2021.728705

**Published:** 2021-08-06

**Authors:** Hans K. Carlson, David C. Vuono, Jennifer B. Glass, Michael W. W. Adams

**Affiliations:** ^1^Lawrence Berkeley National Laboratory, Environmental Genomics and Systems Biology Division, Berkeley, CA, United States; ^2^Department of Civil and Environmental Engineering, Colorado School of Mines, Golden, CO, United States; ^3^Division of Earth and Ecosystem Sciences, Desert Research Institute, Reno, NV, United States; ^4^School of Earth and Atmospheric Sciences, Georgia Institute of Technology, Atlanta, GA, United States; ^5^Department of Biochemistry and Molecular Biology, University of Georgia, Atlanta, GA, United States

**Keywords:** energy metabolism, mechanistic microbial ecology, geochemical constraints, selective pressure, metabolic inhibitors

A mechanistic and predictive understanding of the genotypic and phenotypic controls on microbial element cycling remains a grand challenge in microbiology. While the composition and concentration of carbon sources, electron donors, and electron acceptors are known to influence microbial community structure by selecting for microbial sub-populations with distinct catabolic and respiratory pathways, selective inhibitors and trace nutrient availability modulate the activity of metabolic enzymes. This in turn influences the distribution of microbial sub-populations with distinct metabolic and respiratory traits, which are then structured by complex multi-dimensional environmental gradients that influence the composition, gene content and element cycling activity of microbiomes. While some parameters are known to select for different respiratory activities (e.g., lanthanides as essential nutrients for methanotrophs, molybdate as a specific inhibitor of sulfate reduction, carbon:nitrogen ratio and concentration as a control on the end-products of nitrate respiration), there are others to discover, and demonstrating how selective parameters operate and mediate element cycling across scales requires multi-disciplinary research in both the lab and field.

The *Frontiers* Research Topic, “Selective Controls on Microbial Energy Metabolisms: From the Microscale to the Macroscale” invited articles on the biochemistry, physiology, and ecology of microbial respiratory metabolisms in laboratory and field contexts with a focus on defining the parameters that selectively influence the activity of microbial element cycling. Within the purview of this topic are studies relating microbial metabolic models to element cycling in the field, connecting thermodynamic constraints to the evolution of metabolic pathways, identifying new metabolic inhibitors or trace nutrient dependencies, or defining the mode of action of selective nutrients or inhibitors.

Ultimately, understanding the microbial ecology of element cycling should improve predictions of element fluxes given measurements of geochemistry and microbiome composition and enable targeted interventions to selectively alter outcomes. Lui et al. present a Framework for Integrating Studies for Microbial Ecology or “FICSME” and emphasize that mechanistic microbial ecology needs to connect measurements across scales and eloquently remind us that atomic and molecular scale processes are responsible for driving global element cycles (Lui et al.). As such, predictions from quantitative biochemical rate equations and genetic measurements should be propagable to complex microbiomes and to landscape scale processes.

Other work in this issue exemplifies how specific biogeochemical parameters can have a selective influence on microbiome structure and function. It remains a major challenge to predict how changes in soil and rhizosphere carbon composition influence the recruitment of bacteria on and around plant roots. Chiniquy et al. observed temporal shifts in the composition of microbial communities associated with sorghum roots. To understand the basis for the microbiome dynamics, they measure the influence of individual carbon sources on the composition of low-complexity microbial enrichment cultures from the sorghum rhizosphere and identify several important rhizospheric carbon sources that selectively enrich for *Pseudomonas* (Chiniquy et al.).

Suri et al. investigated changes in denitrification kinetics and intermediate accumulation in response to variable nitrate/nitrite concentrations and pH in *Thauera* sp. from carbon rich oil reservoir environments. The authors' found that decreased pH led to down-regulation of the expression of denitrification genes associated with nitrite and nitrous oxide respiration. Importantly, these results suggest that a possible intervention to achieve complete denitrification in such systems might be to buffer pH through the addition of lime (Suri et al.).

Dulay et al. studied the transcriptional response to cobalt toxicity in the model metal reducing bacterium *Geobacter sulfurreducens* and propose that the relative resistance of *Geobacter* to cobalt may confer a selective advantage in metal-rich environments where dissimilatory metal reduction is a dominant element cycling process (Dulay et al.). Understanding how gradients of alternative metals might influence the relative fitness of dissimilatory metal reduction is important for predicting the prevalence of this metabolism in anoxic systems.

Some work in this issue focuses on characterizing the molecular mechanisms of certain inhibitory compounds. Zane et al. present evidence that in the model sulfate-reducing bacterium *Desulfovibrio vulgaris* Hildenborough, the toxicity of the molybdate is due to a previously unrecognized protein that likely drives an ATP-consuming futile cycle (Zane et al.). This finding brings into question the dominant model that futile cycling of molybdate by the sulfate adenyltransferase is the dominant mode of toxicity of this selective inhibitor of sulfate-reducing bacteria. While the gene identified by Zane et al. is not conserved in all SRM, this work clearly shows the importance of context-specific functional genomic understanding for predicting the mechanism and impact of selective compounds, like molybdate, on microbiomes.

The importance of genotype-metabolic phenotype relationships is also investigated by Peng et al. who report on the relative importance of inositol polyphosphate kinase KCS1 vs. Vip1 in *Candida albicans* (Peng et al.). Mutations in these genes have dramatic consequences for metabolic fluxes between glycolytic, substrate-level phosphorylation, and mitochondrial respiration. This work also speaks to the importance of considering genomic context of these mutations as the authors found that relative toxicity of mutations in these *kcs1* and *vip1* are inverted in *C. albicans* relative to the related yeast in *S. cerevisiae*.

Finally, thermodynamics are fundamental for understanding how geochemical energy landscapes will favor different microbial energy metabolisms. LaRowe et al. discuss the thermodynamic feasibility of various metabolisms associated with the transformation of manganese minerals (LaRowe et al.). As such, this work establishes an important roadmap for the sort of mechanistic studies discussed throughout this issue and suggests new areas of investigation to further understand the environmental conditions that favor manganese metabolic transformation.

The work collected in this topic will contribute to our understanding of how geochemical constraints interact with biological systems to determine element cycling outcomes. We hope that the experimental approaches, findings, and insights will serve as inspiration for future studies and the development of thorough mechanistic and predictive understanding of the selective controls on microbial energy metabolisms.

## Author Contributions

All authors listed have made a substantial, direct and intellectual contribution to the work, and approved it for publication.

## Conflict of Interest

The authors declare that the research was conducted in the absence of any commercial or financial relationships that could be construed as a potential conflict of interest.

## Publisher's Note

All claims expressed in this article are solely those of the authors and do not necessarily represent those of their affiliated organizations, or those of the publisher, the editors and the reviewers. Any product that may be evaluated in this article, or claim that may be made by its manufacturer, is not guaranteed or endorsed by the publisher.

